# Burden of pemphigus vulgaris with a particular focus on women: A review

**DOI:** 10.1097/JW9.0000000000000056

**Published:** 2022-10-03

**Authors:** Nika Kianfar, Shayan Dasdar, Hamidreza Mahmoudi, Maryam Daneshpazhooh

**Affiliations:** a Autoimmune Bullous Diseases Research Center, Tehran University of Medical Sciences, Tehran, Iran

**Keywords:** autoimmune bullous diseases, burden, pemphigus, pemphigus vulgaris, women

## Abstract

Pemphigus vulgaris is a chronic autoimmune mucocutaneous blistering disorder. Apart from the disease itself, other aspects of patients’ life, including psychological, social, and financial, can be affected. Women are particularly more disposed to the impact of the disease due to their physiological characteristics, the specific periods of pregnancy and lactation as well as their social and familial role. In this review, we summarized the burden of pemphigus vulgaris on various aspects of women’s lives. It is essential to understand these problems and provide appropriate support for patients with such a burdensome disease.

**What is known about this subject in regard to women and their families?**

- Pemphigus vulgaris is a chronic blistering disease of the skin and mucous membranes that is expected to further affect women’s lives due to their physiological and psychosocial characteristics.

**What is new from this article as messages for women and their families?**

- Pemphigus vulgaris has more severe effects on women patients in various aspects such as psychological, treatment costs, daily activities, family relationships, and quality of life. They also experience an additional burden due to the possible effect of disease and medications on the fetus in pregnancy and lactation period. Therefore, this point should always be considered by all health care providers to pay special attention to women in the process of decision making.

## Introduction

Pemphigus vulgaris (PV) is a chronic autoimmune blistering disorder of the skin and mucous membranes.^1^ The main immunohistopathologic features of PV are acantholysis and suprabasal cleft as well as IgG and/or C3 intercellular deposits in the epidermis. The presence of anti-desmoglein-3 IgG is mainly responsible for the pathogenesis of PV.^2^

In managing patients with PV, it is essential to know that all the problems these people experience are not merely related to the disease activity. In recent years, a great body of literature has been devoted to examining how this condition would interfere with patients’ normal lives. Women with pemphigus, due to their physiological characteristics and the periods of pregnancy and breastfeeding, would be more vulnerable to the impact of disease than men.^3^ Herein, a brief overview of the studies concerning the effect of PV on various aspects of individual, social, and family life with a particular focus on women is provided.

## Prevalence

In most countries, PV is the predominant form of the pemphigus group.^1^ The incidence of PV varies widely depending on the geographic area and ethnicity.^4^ The annual new cases of PV per million individuals range from 0.5 in Germany,^5^ 0.8 in Finland,^6^ 16.0 in Iran,^7^ 16.1 in Israel,^8^ to 32.0 among Jewish individuals in Connecticut, the United States.^9^ Certain ethnic groups of Ashkenazi Jews and Mediterranean descent are exceptionally predisposed to PV, suggesting genetic susceptibility.^1,10,11^

A female preponderance of pemphigus has been reported, with an average female/male ratio of 1.4:1, ranging between 1.1 in Finland and 5.0 in the United States.^1,10^ However, some exceptions with a male predominance are also available.^12^ The majority of patients are aged 40 to 60 years at the onset of the disease. However, PV may arise at any age, and the onset of disease in some regions like the Middle East was reported to be in younger ages.^10^

## Treatment

Innumerable management strategies have been employed for the treatment of PV. Owing to the recently conducted randomized controlled trials with long-term follow-up data, experts have developed comprehensive guidelines for disease management.^13,14^

Corticosteroids (CSs), usually in the form of oral predniso(lo)ne, with or without adjuvant therapies, are recommended as first-line therapy.^15^ The evidence for the optimal CS dose is yet to be delineated, but oral predniso(lo)ne 1 mg/kg daily is generally used, which should be tapered at the end of the consolidation phase.^16,17^ The adjuvant therapies supported by the literature are immunosuppressive agents, like azathioprine, mycophenolate mofetil (MMF), cyclophosphamide, and rituximab (RTX). The use of other conventional immunosuppressives, including methotrexate and cyclosporine, was not recommended in recent guidelines.^13,16^

Following the study of Joly et al.^18^ showing the superiority of RTX plus prednisone versus prednisone alone, RTX has been designated by the European Medicines Agency and the US Food and Drug Administration as the first-line therapy of adults with moderate-to-severe PV.^16^ Werth et al.^19^ also demonstrated that RTX acted better than MMF in inducing sustained complete remission in PV. More recently, various case series also reported the possible use of RTX in vulnerable patients.^20–23^

Azathioprine (AZT) (2 mg/kg/d) and MMF (2 g/d) were the most commonly used first-line CS-sparing agents in PV before identifying the efficacy of RTX.^16,17^ Nowadays, they are prescribed when RTX is contraindicated or lacks insurance coverage.^15,24^ Finally, intravenous immunoglobulin (IVIg), plasmapheresis, or immunoadsorption are other options for treating severe or refractory PV.^25–27^

## Adverse events

Although CSs have dramatically improved PV prognosis, they come with various well-documented cutaneous, metabolic, and systemic complications.^28^ Postmenopausal women are the group most vulnerable to primary osteoporosis, and CSs can expose them to higher risk.^29^ The chronic use of CSs in women can also harm reproductive function. CSs affect the ovary by suppressing the hypothalamo-pituitary-gonadal axis or directly connecting to its receptors. Patients may experience menstrual difficulties in the form of amenorrhea and postmenopausal bleeding.^30^ Infertility is also reported in women with pemphigus.^31^ RTX is generally a safe treatment option, and its most accompanied complications are infusion-related adverse events, which are mild and transient. On the other hand, RTX makes patients susceptible to opportunistic infections.^32,33^ Both AZT and MMF decrease the reliance on CSs; however, they carry the risk of hematologic abnormalities, hepatotoxicity, and opportunistic infections.^17,34,35^

## Pregnancy

Women with PV may desire to become pregnant, but their underlying disorder would become a major impediment in their way. Like many other autoimmune disorders, PV course may exacerbate during pregnancy and postpartum with reported rates of 54% and 44%, respectively.^36^ Several factors might fluctuate pemphigus status during pregnancy. Hormonal changes, including estrogen and progesterone as well as cortisol, norepinephrine, and dehydroepiandrosterone, occur to accommodate the fetus.^37^ These hormonal alterations evoke shifting of naive T cells toward more T helper (Th) 2 immune response in order not to reject the fetus.^38^ The disturbance of the Th1:Th2 balance may trigger or exacerbate Th2-mediated autoimmune diseases, such as pemphigus, during pregnancy.^39,40^ In addition to this immunological pathway, women’s concern for the potential harm of medications to their pregnancy may affect their adherence to the treatment.^41^ Although no estimate of drug cessation during pregnancy in patients with pemphigus exists, it may be an important factor affecting disease exacerbation in pregnancy. Some women may also develop new lesions induced by the trauma of cesarean section and breastfeeding, as a form of Köbner phenomenon.^42^

Poor control of maternal PV may result in some adverse pregnancy outcomes, including preterm birth, spontaneous abortion, stillbirth, and occasionally neonatal pemphigus.^43^ Pregnant women with active PV can passively transfer autoantibodies to their neonate, causing transient blisters of the skin and rarely the mucous membranes of the neonates. Neonatal PV usually spontaneously resolves within 1 to 4 weeks without special treatment. Noteworthy is that if the disease is well-controlled, most women with pemphigus would give birth to a term, healthy baby.^43–45^

A great challenging issue in women with PV is the choice of medication due to safety concerns during pregnancy and lactation. CSs are considered the treatment of choice in PV in this period.^46,47^ CSs can be used alone or combined with other immunosuppressive drugs such as AZT, IVIg, or plasmapheresis in refractory disease.^46^ Data regarding paternal exposure to immunosuppressive agents are limited. Although there were concerns regarding the use of MMF and methotrexate, recent studies reported no increased risk of infertility and adverse pregnancy outcomes in fathers taking immunosuppressives.^48,49^ Table 1 outlines the key points of therapeutic plans during pregnancy and breastfeeding. Given these circumstances, it is recommended to use family planning and contraceptive measures until their disease is well controlled. Medical consultation of PV during pregnancy requires collaboration among dermatologists, obstetricians, and pediatricians to control the disease. They should also reassure the mother that their treatments are safe causing no harm to the fetus’s health.

**Table 1:** Key points of the management of pemphigus vulgaris during pregnancy and lactation^38,44,45^

Agent	FDA pregnancy category	Compatible with pregnancy	Compatible with breastfeeding	Comments
Systemic CSs	C	Safe Unwanted complications like diabetes, hypertension, intrauterine growth restriction, premature rupture of membranes, and orofacial clefts may occur.	Safe	The daily dose should not exceed 20 mg of prednisolone. Breastfeed at least 4 hr after consumption.
Topical CSs	C	Safe	Safe Transmission through breastmilk is negligible.	High-potent topical steroids should be avoided. Breastfeed at least 4 hr after application around the nipple area.
Rituximab	C	Not recommended. The placental passage increases from the first to the last trimester. The neonates of mothers who were receiving RTX showed no increased risk of infection, but prematurity and spontaneous abortion have been reported.	Not recommended. Transmission through breast milk is minimal.	Women are advised to conceive at least 12 mo after the last RTX infusion according to drug leaflet. Due to the lack of safety evidence, women should not breastfeed while treated with this drug and for at least 6 mo after.
Azathioprine	D	Safe. AZT was previously accused of causing congenital anomalies; however, recent studies found no increased risk of the drug to the mother and her fetus.	Safe. Transmission through breastmilk is negligible.	The daily dose should not exceed 2 mg/kg/d. Breastfeed at least 4 hr after consumption.
Mycophenolate compound	D	Contraindicated. Teratogenic	Contraindicated. Transmission through breastmilk is significant.	Must be stopped and replaced 6 wk prior to conception in women.
Intravenous IVIg	C	Safe. The neonates of mothers who were receiving IVIg showed no increased risk of infection or major adverse event.	Safe.	Reserve for severe or recalcitrant PV.
Plasmapheresis or immunoadsorption		Safe. The neonates of mothers who were receiving plasmapheresis or immunoadsorption showed no increased risk of infection or major adverse event.	Safe.	Reserve for severe or recalcitrant PV.
Cyclophosphamide	D	Contraindicated. Teratogenic	Contraindicated. Transmission through breast milk is significant	Must be stopped and replaced 1 mo prior to conception.

CSs, corticosteroids; FDA, US Food and Drug Administration; IVIg, immunoglobulin; RTX, rituximab; AZT, azathioprine.

## Psychosocial burden

Pemphigus is a debilitating disorder that predisposes patients to a high risk of psychological problems.^50^ A recent systematic review has shown that the prevalence of depressive symptoms and clinical depression among patients with autoimmune bullous disorders (AIBDs) ranged from 40 to 80% and 11.4 to 28%, respectively.^51^ Wohl et al.^52^ evaluated records of 764 individuals in Israel (255 pemphigus patients and 509 controls) and found that depression was higher in pemphigus patients with an odds ratio of 1.19 (95% confidence interval (CI): 1.12-1.27). Similarly, Hsu et al.^53^ analyzed 4600 medical records in Taiwan (926 pemphigus patients and 3674 controls) and reported that pemphigus patients were more likely to suffer from depression with an adjusted hazard ratio of 1.99 (95% CI: 1.37-2.86).^53^ Smaller cross-sectional studies also reported mental health impairment prevalence of 74% in Iran,^54^ 59% in Egypt,^55^ 47% in Korea,^56^ 40% in Italy,^57^ and 40% in India.^58^ Other less-documented psychiatric disorders with higher prevalence among pemphigus patients than healthy individuals are schizophrenia,^59^ bipolar disorder,^60^ obsessive-compulsive disorder,^54^ and post-traumatic stress disorder.^61^

Some studies have tried to figure out factors that predispose pemphigus patients to mental health challenges. Accordingly, Hsu et al.^53^ reported that the female gender appears to increase the risk of depression in patients with PV. Regarding steroid-induced psychiatric reactions, the female gender was considered as a risk factor as well.^62^ Although this gender disparity was not further investigated to detect the role of social, biological, and hormonal factors, it still suggests the vulnerability of this population. The disease activity is another well-supported predisposing factor for psychiatric syndromes.^54,56–58,63^ However, even in the quiescent periods of disease, the psychological burden of the disease remains quite high, specifically for women.^64,65^ Evaluating psychological morbidity of pemphigus patients who were in clinical remission demonstrated a prevalence of depression and anxiety of 30.8 and 35.5%, respectively.^66^ The duration of active illness and hospitalization are among the factors affecting psychological symptoms in the remission phase of the disease.^66^ Therefore, it is not surprising that RTX therapy with rapid and prolonged therapeutic effect could improve pemphigus patients’ mental well-being.^67^

The association of pemphigus and psychological disorders seems bi-directional as stressful life events can initiate or worsen course of pemphigus.^68,69^ A study on AIBDs have revealed a higher odds of disease development among individuals with history of depression and anxiety and importantly with greater probability in women.^70^

To date, no agreement has been reached on why psychological symptoms are so prevalent in pemphigus patients, although there are some probable etiologies. First of all, PV and its treatments, specifically systemic CSs, are associated with a disfiguring and cushingoid appearance that might cause social stigma and troublesome changes in patients’ lifestyles.^71^ Second, current evidence indicated that proinflammatory cytokines are partially involved in developing psychosocial disorders.^72,73^ Several observations have recently highlighted the association of mental health challenges with autoimmune conditions.^74,75^ Finally, long-term immunosuppressive therapy with CSs may cause disturbance in psychiatric health (depression, mania, and cognitive impairment), known as the most common adverse event of steroids according to the Glucocorticoid Toxicity Index.^28^

The data regarding the relationship between pemphigus and psychiatric disorders is still in its infancy. Further studies are needed to determine the pathophysiology of this association to lessen the psychological burden of the disease. Clinicians should be more careful about the psychological symptoms in patients with pemphigus, especially women, to promptly diagnose and request a necessary therapeutic consult. In addition, ongoing support from family, friends, and society should be provided for these patients to optimize treatment outcomes.

## Economic burden

Neglecting the possible economic burden of pemphigus could have detrimental consequences for patients and society.^76^ This issue becomes even more prominent in women, as they are often not financially independent, especially in developing countries.

A cohort national-based survey that tracked the inpatient financial burden of pemphigus using the largest available health expenditure database in the United States demonstrated a mean annual cost of $14,520 for patients with a primary diagnosis of pemphigus and $9,948 for patients with any other diagnosis apart from pemphigus.^77^ More importantly, they detected a higher rate of hospitalization, length, and cost of stay in the nonwhite race, poor, and underinsured/uninsured patients, which indicated the presence of disparities for minorities in pemphigus care. The female gender was also found to have an odds ratio of 1.10 (95% CI: 1.01-1.20) for admission compared with male patients. The high medical utilization for pemphigus patients compared to other diseases was also highlighted in other societies in Taiwan^78^ and Germany.^79^ Evaluating the cost of illness from a social perspective illustrated that indirect costs are even higher than direct costs. Brodszky et al.^80^ reported that obscure items of health care costs, including productivity loss and informal care, have 75% of the associated costs with pemphigus, which was significantly lower among women.

The introduction of new biological agents such as RTX and IVIg was a shifting paradigm in treating pemphigus patients. Despite the great improvement in the prognosis of patients with pemphigus, there have always been concerns about the high cost of these medications.^81^ A pharmacoeconomic study on patients with PV found that IVIg therapy would approximately halve the long-term cost compared to conventional therapy.^82^ Similarly, RTX, which is even cheaper than IVIg, results in an effective decrease in health care costs, owing to reducing admissions, length of stay, and treatment-related adverse events.^83–85^

## Delay in diagnosis

The prompt diagnosis of pemphigus is of utmost importance to disrupt disease progression and improve prognosis, but unfortunately, many patients experience long delays to diagnosis.^86,87^ This frustrating undiagnosed period is often due to a lack of health care providers’ knowledge of pemphigus manifestations, long waits for a dermatologist visit, and repeated inconclusive diagnostic tools. This ineffective passage of time makes patients feel they have no control over their bodies and escalates their concerns. Consequently, some of them may decide to change their doctors, which means starting this inconvenient process all over again.^88^

This delay in diagnosis is more or less constant among different reports; accordingly, there is an average 6 months lag from the first presentation to definite diagnosis.^89–92^ It is shown that pemphigus in the oral cavity is diagnosed later than on the skin due to more non-specific features of mucosal involvement.^89,91,93^ A survey conducted in the United States, employing the International Pemphigus and Pemphigoid Foundation disease registry system to 393 PV patients, revealed a longer period of diagnosis in women as they were more inclined toward mucosal involvement.^93^

Most patients with pemphigus are not satisfied with the level of information shared with them. They believe that if they get proper attention and meet their expectations, this long period would be more tolerable.^88^ Therefore, in addition to attempts to reduce this lengthy diagnostic period, physicians should better understand patients’ conditions.

## Daily activities

Many patients with PV experience extreme restrictions in their daily activities.^94^ Due to probable stigmatization in the workplace, productivity loss is not far-fetched.^95^ To quantify this phenomenon, Wang et al.^96^ utilized the Work Productivity and Activity Impairment Questionnaire-Specific Health Problem, a specific tool to measure productivity loss. They found that pemphigus patients with poor treatment response had significantly greater work and activity impairment than those with a proper response. Besides, approximately 15% of patients were stigmatized at work leading to the resignation of 1 in every 3.^96^ Sleep disturbance is another frequent problematic condition for patients with pemphigus, with an odds ratio of 18.02 (95% CI: 2.46-131.88) compared to healthy individuals,^97^ which can be worsened by the use of CSs.^98^ Patients with lesions in the oral cavity might also experience eating difficulties and alterations in dietary habits.^99^

Pemphigus could also alter patients’ engagement with leisure activities. This change largely relies on their social characteristics, not financial and facility status.^100^ Encouraging patients to participate in suitable physical activities could be a big move toward a normal life.^101^

## Familial relationships

Pemphigus could result in patients’ discomfort in domestic and social relationships. Although genital involvement is not rare in women, it may go unnoticed during the examination and cause dyspareunia in many cases.^102^ Interviewing 10 patients with PV, Piri et al.^103^ documented various familial challenges owing to a lack of knowledge of the disease. A single woman reported the loss of a chance for marriage; another one was divorced after genital involvement and cessation of sexual relationship. Besides, several patients complained about isolation due to fear of affecting others. Therefore, a complete pelvic examination is recommended to provide the appropriate medical treatments and Pap smear when genital involvement exists.^102^

For evaluating the impact of pemphigus on family members, Family Dermatology Life Quality Index is a useful validated tool. Accordingly, pemphigus caused a remarkable impairment in the quality of life (QoL) of patients’ families, associated with disease severity, mucocutaneous involvement, shorter disease duration, and male sex of patients that worsened by older age, low education, and the marital status of caregivers.^104,105^ Improving knowledge and problem-solving abilities in patients and families can greatly reduce the disease burden.^106^

## QoL instruments and measurements

Several assessment tools have been utilized to evaluate the QoL of patients with PV. Some of these instruments were general for any illness, and some were related to skin disorders. Specific tools for AIBDs were also issued in recent years. Herein, a concise review of various studies with different methods of QoL evaluation is provided.

## Generic instrument

The Medical Outcome Study 36-item Short-form Survey is the most frequently used questionnaire among the general QoL assessing tools. Terrab et al.,^107^ by employing this questionnaire, found great alterations in QoL of pemphigus patients in Morocco, especially women, due to the importance of self-image and various social and marital consequences. From Italy, Tabolli et al.^63^ reported mucocutaneous PV involvement as the most burdensome clinical subtype. Moreover, their subsequent investigation emphasized greater health impairment in women and older patients.^57^ A meta-analysis of studies that utilized Medical Outcome Study 36-item Short-form Survey from different regions demonstrated that the most prominent health deterioration is caused in physical- and emotional-role domains.^107,108^

## Skin-related instrument

For a more specific measure of QoL based on skin symptoms, in several studies, dermatology-specific metrics such as the DLQI were used. One of the first reports on the use of DLQI in blistering disorders was from Germany, recording a mean score of 10 in patients with PV, which was worse than other skin conditions.^109^ Subsequent studies also indicated a low QoL in pemphigus patients using this instrument with a mean DLQI score of 10.9 in Iran,^110^ 10.2 in Korea,^56^ and 5.4 in Hungary.^111^ In these studies, several factors were recognized to increase DLQI score, including longer disease duration, mucocutaneous phenotype, itching, positive Nikolsky’s sign, and CS dose; but what consistently deteriorated QoL was the severity of the disease. Notably, gender was not found to affect DLQI score in these studies.^56,105,110,111^

## AIBD-specific instruments

Autoimmune Bullous Disease Quality of Life (ABQOL) is a more sensitive tool for AIBDs to capture small changes in disease status. In the study conducted by Murrell et al. to assess the validity and reliability of the questionnaire, the authors reported that in patients with PV, the mean ABQOL score was 11.5, and associated poorly with Pemphigus Disease Area Index.^112^ The reported mean ABQOL score for PV patients in other studies were as follows: 16.4 in the United States,^113^ 17.4 in Poland,^114^ 17.3 in China,^115^ about 16 in Egypt/Tunisia,^116^ and 29.4 in Iran.^117^ Regarding the association of ABQOL with Pemphigus Disease Area Index score, those studies have made various comments, from no correlation^116^ to poor^114^ and moderate correlation.^118,119^

Noteworthy, while most patients in those studies were in their remission phase of the disease, only patients with active disease were enrolled in the study from Iran, which explains the higher score in their study. They found significantly lower QoL in women (ABQOL: 31.0) compared to men (ABQOL: 27.7), which was not reported in other studies.^117^

To differentiate treatments effect from the disease itself, Treatment of Autoimmune Bullous Disease Quality of Life (TABQOL) was designed.^120^ Based on the available literature, the TABQOL score of pemphigus patients would change according to disease course^114,116^ and treatment intensity,^120,121^ but unexpectedly, the difference between various treatments^119,122^ and time periods^118,119^ was not remarkable. Moreover, no study indicated the significant effect of gender on TABQOL score.

## Take away points and conclusions

**Fig. 1.**
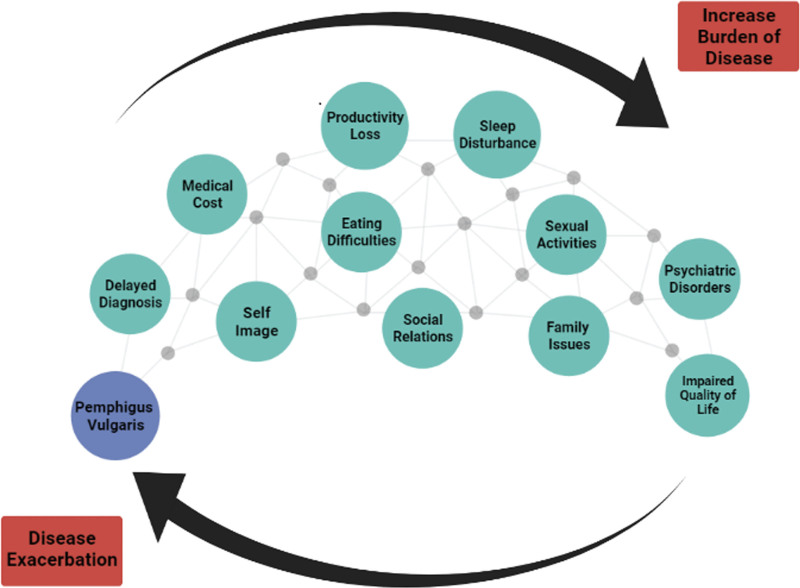
Problems derived from pemphigus vulgaris are interrelated, leading to an increased burden of disease in one way and disease exacerbation in another way.

As discussed above, PV would affect different aspects of patient’s life. Many of these problems are interconnected and can also worsen the disease either directly or indirectly. Therefore, underestimating these problems, in addition to the augmented burden on patients, could lead to disease exacerbation (Fig. 1). Women have a unique set of difficulties to deal with, such as psychological problems, social and family relationships, and financial issues. Besides, they have specific worries in pregnancy and breastfeeding due to both the alternation in the disease condition and the fear of the medications’ effect on the fetus/infant. All indicate that women often endure a more disturbed QoL.

To diminish the disease burden, a multidisciplinary approach is required with the following goals: (a) support patients with healthcare costs by modification at the health legislation, (b) increase general health providers’ knowledge of disease for earlier diagnosis, (c) establish support groups to revive patients’ social relationships and mental health, and (d) increase patients’ and their families’ awareness of PV.

## Conflicts of interest

None.

## Funding

None.

## Author contributions

M.D. and H.M. conceived and supervised the study; N.K. and S.D. performed the literature review and wrote the manuscript; all authors approved the final manuscript.

## Study approval

N/A.

## References

[1] Porro AM Seque CA Ferreira MCC. Pemphigus vulgaris. An Bras Dermatol 2019;94:264–78. 31365654 10.1590/abd1806-4841.20199011 PMC6668932

[2] Egami S Yamagami J Amagai M. Autoimmune bullous skin diseases, pemphigus and pemphigoid. J Allergy Clin Immunol 2020;145:1031–47. 32272980 10.1016/j.jaci.2020.02.013

[3] Zhao CY Murrell DF. Autoimmune blistering diseases in females: a review. Int J Women’s Dermatol 2015;1:4–12. 28491949 10.1016/j.ijwd.2015.01.002 PMC5418673

[4] Alpsoy E Akman-Karakas A Uzun S. Geographic variations in epidemiology of two autoimmune bullous diseases: pemphigus and bullous pemphigoid. Arch Dermatol Res 2015;307:291–8. 25589418 10.1007/s00403-014-1531-1

[5] Bertram F Bröcker EB Zillikens D. Prospective analysis of the incidence of autoimmune bullous disorders in Lower Franconia, Germany. J Dtsch Dermatol Ges 2009;7:434–40. 19170813 10.1111/j.1610-0387.2008.06976.x

[6] Hietanen J Salo OP. Pemphigus: an epidemiological study of patients treated in Finnish hospitals between 1969 and 1978. Acta Derm Venereol 1982;62:491–6. 6187153

[7] Chams-Davatchi C Valikhani M Daneshpazhooh M. Pemphigus: analysis of 1209 cases. Int J Dermatol 2005;44:470–6. 15941433 10.1111/j.1365-4632.2004.02501.x

[8] Pisanti S Sharav Y Kaufman E. Pemphigus vulgaris: incidence in Jews of different ethnic groups, according to age, sex, and initial lesion. Oral Surg Oral Med Oral Pathol 1974;38:382–7. 4528670 10.1016/0030-4220(74)90365-x

[9] Simon DG Krutchkoff D Kaslow RA. Pemphigus in Hartford County, Connecticut, from 1972 to 1977. Arch Dermatol 1980;116:1035–7. 7416755

[10] Kridin K Schmidt E. Epidemiology of pemphigus. JID Innovations 2021;1:100004. 34909708 10.1016/j.xjidi.2021.100004 PMC8659392

[11] Ahmed AR Yunis EJ Khatri K. Major histocompatibility complex haplotype studies in Ashkenazi Jewish patients with pemphigus vulgaris. Proc Natl Acad Sci USA 1990;87:7658–62. 2217197 10.1073/pnas.87.19.7658 PMC54807

[12] Kutlubay Z Sevim Keçici A Çelik U. A survey of bullous diseases in a Turkish university hospital: clinicoepidemiological characteristics and follow-up. Turk J Med Sci 2021;51:124–33. 32892539 10.3906/sag-2006-231 PMC7991873

[13] Joly P Horvath B Patsatsi Α. Updated S2K guidelines on the management of pemphigus vulgaris and foliaceus initiated by the European Academy of Dermatology and Venereology (EADV). J Eur Acad Dermatol Venereol 2020;34:1900–13. 32830877 10.1111/jdv.16752

[14] Schmidt E Kasperkiewicz M Joly P. Pemphigus. Lancet 2019;394:882–94. 31498102 10.1016/S0140-6736(19)31778-7

[15] Bilgic A Murrell DF. What is novel in the clinical management of pemphigus. Expert Rev Clin Pharmacol 2019;12:973–80. 31550941 10.1080/17512433.2019.1670059

[16] Murrell DF Peña S Joly P. Diagnosis and management of pemphigus: recommendations of an international panel of experts. J Am Acad Dermatol 2020;82:575–85.e1. 29438767 10.1016/j.jaad.2018.02.021 PMC7313440

[17] Didona D Maglie R Eming R. Pemphigus: current and future therapeutic strategies. Front Immunol 2019;10:1418. 31293582 10.3389/fimmu.2019.01418 PMC6603181

[18] Joly P Maho-Vaillant M Prost-Squarcioni C. First-line rituximab combined with short-term prednisone versus prednisone alone for the treatment of pemphigus (Ritux 3): a prospective, multicentre, parallel-group, open-label randomised trial. Lancet 2017;389:2031–40. 28342637 10.1016/S0140-6736(17)30070-3

[19] Werth VP Joly P Mimouni D. Rituximab versus mycophenolate mofetil in patients with pemphigus vulgaris. N Engl J Med 2021;384:2295–305. 34097368 10.1056/NEJMoa2028564

[20] Kianfar N Dasdar S Mahmoudi H. Rituximab in childhood and juvenile autoimmune bullous diseases as first-line and second-line treatment: a case series of 13 patients. J Dermatolog Treat 2020;1:6. 10.1080/09546634.2020.1788702 32589481

[21] Patel MH Brumfiel CM Bohrer N. Efficacy of rituximab in pediatric pemphigus: a literature review. JAAD Int 2022;6:6–10. 34825231 10.1016/j.jdin.2021.10.002 PMC8605268

[22] Vassallo C Grassi S Tagliabue E. Pregnancy outcome after rituximab treatment before conception in patients affected by severe pemphigus vulgaris/superficialis. J Eur Acad Dermatol Venereol 2017;31:e331–3. 28079925 10.1111/jdv.14119

[23] Dehghanimahmoudabadi A Kianfar N Akhdar M. Pregnancy outcomes in women with pemphigus exposed to rituximab before or during pregnancy. Int J Women’s Dermatol 2022;8:e038. 35837335 10.1097/JW9.0000000000000038 PMC9276145

[24] Malik AM Tupchong S Huang S. An updated review of pemphigus diseases. Medicina (Kaunas) 2021;57:1080. 34684117 10.3390/medicina57101080 PMC8540565

[25] Tan-Lim R Bystryn JC. Effect of plasmapheresis therapy on circulating levels of pemphigus antibodies. J Am Acad Dermatol 1990;22:35–40. 2298963 10.1016/0190-9622(90)70004-2

[26] Amagai M Ikeda S Shimizu H. A randomized double-blind trial of intravenous immunoglobulin for pemphigus. J Am Acad Dermatol 2009;60:595–603. 19293008 10.1016/j.jaad.2008.09.052

[27] Kridin K. Emerging treatment options for the management of pemphigus vulgaris. Ther Clin Risk Manag 2018;14:757–78. 29740210 10.2147/TCRM.S142471 PMC5931200

[28] Liang Y Zeng FAP Sheriff T. Evaluation of the toxicity of glucocorticoids in patients with autoimmune blistering disease using the Glucocorticoid Toxicity Index: a cohort study. JAAD Int 2022;6:68–76. 35059661 10.1016/j.jdin.2021.09.003 PMC8760348

[29] Lupsa BC Insogna KL Micheletti RG. Corticosteroid use in chronic dermatologic disorders and osteoporosis. Int J Women’s Dermatol 2021;7:545–51. 35024411 10.1016/j.ijwd.2021.07.014 PMC8721058

[30] Khizroeva J Nalli C Bitsadze V. Infertility in women with systemic autoimmune diseases. Best Pract Res Clin Endocrinol Metab 2019;33:101369. 31837981 10.1016/j.beem.2019.101369

[31] Ouahes N Qureshi TA Ahmed AR. Infertility in women with pemphigus vulgaris and other autoimmune diseases. J Am Acad Dermatol 1997;36:383–7. 9091468 10.1016/s0190-9622(97)80213-7

[32] Tavakolpour S Mahmoudi H Balighi K. Sixteen-year history of rituximab therapy for 1085 pemphigus vulgaris patients: a systematic review. Int Immunopharmacol 2018;54:131–8. 29132070 10.1016/j.intimp.2017.11.005

[33] Kasperkiewicz M Eming R Behzad M. Efficacy and safety of rituximab in pemphigus: experience of the German Registry of Autoimmune Diseases. J Dtsch Dermatol Ges 2012;10:727–32. 22577946 10.1111/j.1610-0387.2012.07931.x

[34] Meggitt SJ Anstey AV Mohd Mustapa MF. British Association of Dermatologists’ guidelines for the safe and effective prescribing of azathioprine 2011. Br J Dermatol 2011;165:711–34. 21950502 10.1111/j.1365-2133.2011.10575.x

[35] Meurer M. Immunosuppressive therapy for autoimmune bullous diseases. Clin Dermatol 2012;30:78–83. 22137230 10.1016/j.clindermatol.2011.03.013

[36] Daneshpazhooh M Chams-Davatchi C Valikhani M. Pemphigus and pregnancy: a 23-year experience. Indian J Dermatol Venereol Leprol 2011;77:534. 10.4103/0378-6323.82404 21727712

[37] Feliciani C Genovese G D’Astolto R. Autoimmune bullous diseases during pregnancy: insight into pathogenetic mechanisms and clinical features. G Ital Dermatol Venereol 2019;154:256–62. 30375213 10.23736/S0392-0488.18.06153-9

[38] Tavakolpour S Mirsafaei HS Delshad S. Management of pemphigus disease in pregnancy. Am J Reprod Immunol 2017;77. 10.1111/aji.12601 27862562

[39] Tavakolpour S Rahimzadeh G. New insights into the management of patients with autoimmune diseases or inflammatory disorders during pregnancy. Scand J Immunol 2016;84:146–9. 27300757 10.1111/sji.12453

[40] Fagundes PPS Santi CG Maruta CW. Autoimmune bullous diseases in pregnancy: clinical and epidemiological characteristics and therapeutic approach. An Bras Dermatol 2021;96:581–90. 34304937 10.1016/j.abd.2020.10.007 PMC8441454

[41] Matsui D. Adherence with drug therapy in pregnancy. Obstetr Gynecol Int 2012;2012:796590. 10.1155/2012/796590 PMC3253470 22242026

[42] Daneshpazhooh M Fatehnejad M Rahbar Z. Trauma-induced pemphigus: a case series of 36 patients. JDDG: Journal der Deutschen Dermatologischen Gesellschaft 2016;14:166–71. 26819113 10.1111/ddg.12738

[43] Kardos M Levine D Gürcan HM. Pemphigus vulgaris in pregnancy: analysis of current data on the management and outcomes. Obstet Gynecol Surv 2009;64:739–49. 19849866 10.1097/OGX.0b013e3181bea089

[44] Lin L Zeng X Chen Q. Pemphigus and pregnancy. Analysis and summary of case reports over 49 years. Saudi Med J 2015;36:1033–8. 26318458 10.15537/smj.2015.9.12270 PMC4613625

[45] Drenovska K Darlenski R Kazandjieva J. Pemphigus vulgaris and pregnancy. Skinmed 2010;8:144–9. 21137619

[46] Kushner CJ Concha JSS Werth VP. Treatment of autoimmune bullous disorders in pregnancy. Am J Clin Dermatol 2018;19:391–403. 29392620 10.1007/s40257-018-0342-0

[47] Genovese G Derlino F Berti E. Treatment of autoimmune bullous diseases during pregnancy and lactation: a review focusing on pemphigus and pemphigoid gestationis. Front Pharmacol 2020;11:583354. 33117178 10.3389/fphar.2020.583354 PMC7566587

[48] Perez-Garcia LF Dolhain R Vorstenbosch S. The effect of paternal exposure to immunosuppressive drugs on sexual function, reproductive hormones, fertility, pregnancy and offspring outcomes: a systematic review. Hum Reprod Update 2020;26:961–1001. 32743663 10.1093/humupd/dmaa022 PMC7600290

[49] Meserve J Luo J Zhu W. Paternal exposure to immunosuppressive and/or biologic agents and birth outcomes in patients with immune-mediated inflammatory diseases. Gastroenterology 2021;161:107–15.e3. 33744307 10.1053/j.gastro.2021.03.020 PMC8238837

[50] Matthews R Ali Z. Comorbid mental health issues in patients with pemphigus vulgaris and pemphigus foliaceus. Clin Exp Dermatol 2022;47:24–9. 34459019 10.1111/ced.14916

[51] Pourali SP Gutierrez Y Kohn AH. Bullous dermatoses and depression: a systematic review. JAMA Dermatol 2021;157:1487–95. 34668929 10.1001/jamadermatol.2021.4055

[52] Wohl Y Mashiah J Kutz A. Pemphigus and depression comorbidity: a case control study. Eur J Dermatol 2015;25:602–5. 26553704 10.1684/ejd.2015.2649

[53] Hsu YM Fang HY Lin CL. The risk of depression in patients with pemphigus: a nationwide cohort study in Taiwan. Int J Environ Res Public Health 2020;17:1983. 10.3390/ijerph17061983 PMC7142767 32192212

[54] Arbabi M Ghodsi Z Mahdanian A. Mental health in patients with pemphigus: an issue to worth consideration. Indian J Dermatol 2011;56:541–5. 22121274 10.4103/0019-5154.87151 PMC3221219

[55] Metwally D Fawzy M ElKalioby M. Assessment of the quality of life, prevalence of depression, and the level of interleukin 6 in patients with pemphigus vulgaris. Acta Dermatovenerol Croat 2020;28:57–62. 32876029

[56] Sung JY Roh MR Kim SC. Quality of life assessment in korean patients with pemphigus. Ann Dermatol 2015;27:492–8. 26512162 10.5021/ad.2015.27.5.492 PMC4622882

[57] Paradisi A Sampogna F Di Pietro C. Quality-of-life assessment in patients with pemphigus using a minimum set of evaluation tools. J Am Acad Dermatol 2009;60:261–9. 19004524 10.1016/j.jaad.2008.09.014

[58] Kumar V Mattoo SK Handa S. Psychiatric morbidity in pemphigus and psoriasis: a comparative study from India. Asian J Psychiatr 2013;6:151–6. 23466113 10.1016/j.ajp.2012.10.005

[59] Kridin K Zelber-Sagi S Comaneshter D. Association between schizophrenia and an autoimmune bullous skin disease-pemphigus: a population-based large-scale study. Epidemiol Psychiatr Sci 2019;28:191–8. 28942756 10.1017/S204579601700052X PMC6998929

[60] Kridin K Zelber-Sagi S Comaneshter D. Bipolar disorder associated with another autoimmune disease-pemphigus: a population-based study. Can J Psychiatry 2018;63:474–80. 29108425 10.1177/0706743717740344 PMC6099770

[61] Wei EX Li SJ Mostaghimi A. Post-traumatic stress disorder in patients with autoimmune blistering diseases. Br J Dermatol 2020;182:1044–5. 31545508 10.1111/bjd.18548

[62] Kenna HA Poon AW de los Angeles CP. Psychiatric complications of treatment with corticosteroids: review with case report. Psychiatry Clin Neurosci 2011;65:549–60. 22003987 10.1111/j.1440-1819.2011.02260.x

[63] Tabolli S Mozzetta A Antinone V. The health impact of pemphigus vulgaris and pemphigus foliaceus assessed using the Medical Outcomes Study 36-item short form health survey questionnaire. Br J Dermatol 2008;158:1029–34. 18294312 10.1111/j.1365-2133.2008.08481.x

[64] Tabolli S Pagliarello C Paradisi A. Burden of disease during quiescent periods in patients with pemphigus. Br J Dermatol 2014;170:1087–91. 24428431 10.1111/bjd.12836

[65] De D Kumar S Handa S. Psychological morbidity in pemphigus patients in clinical remission and its relation with clinico-demographic parameters. Journal der Deutschen Dermatologischen Gesellschaft = Journal of the German Society of Dermatology: JDDG 2021;20:26–33. 34821016 10.1111/ddg.14605

[66] De D Kumar S Handa S. Psychological morbidity in pemphigus patients in clinical remission and its relation with clinico-demographic parameters. Journal der Deutschen Dermatologischen Gesellschaft = Journal of the German Society of Dermatology: JDDG 2022;20:26–33. 10.1111/ddg.14605 34821016

[67] Rashid H Poelhekken M Meijer JM. The positive impact of rituximab on the quality of life and mental health in patients with pemphigus. JAAD Int 2022;7:31–3. 35252887 10.1016/j.jdin.2022.01.004 PMC8892131

[68] Morell-Dubois S Carpentier O Cottencin O. Stressful life events and pemphigus. Dermatology 2008;216:104–8. 18216471 10.1159/000111506

[69] Cremniter D Baudin M Roujeau JC. Stressful life events as potential triggers of pemphigus. Arch Dermatol 1998;134:1486–1487. 9828894 10.1001/archderm.134.11.1486

[70] Kridin K Hundt JE Ludwig RJ. Anxiety and depression predispose individuals to an autoimmune bullous diseases- bullous pemphigoid: a large-scale population-based cohort study. Curr Psychol 2021.

[71] Mazzotti E Mozzetta A Antinone V. Psychological distress and investment in one’s appearance in patients with pemphigus. J Eur Acad Dermatol Venereol 2011;25:285–9. 20626535 10.1111/j.1468-3083.2010.03780.x

[72] Himmerich H Patsalos O Lichtblau N. Cytokine research in depression: principles, challenges, and open questions. Front Psychiatry 2019;10:30. 30792669 10.3389/fpsyt.2019.00030 PMC6374304

[73] Vogelzangs N de Jonge P Smit JH. Cytokine production capacity in depression and anxiety. Transl Psychiatry 2016;6:e825. 27244234 10.1038/tp.2016.92 PMC5070051

[74] Gregory JM Mak M McIntyre RS. Chapter 21 - Inflammation and depression in patients with autoimmune disease, diabetes, and obesity. In: Baune BT, editor. Inflammation and Immunity in Depression. Academic Press 2018. p. 377–92.

[75] Wang LY Chiang JH Chen SF. Systemic autoimmune diseases are associated with an increased risk of bipolar disorder: a nationwide population-based cohort study. J Affect Disord 2018;227:31–7. 29049933 10.1016/j.jad.2017.10.027

[76] Stammen LA Stalmeijer RE Paternotte E. Training physicians to provide high-value, cost-conscious care: a systematic review. JAMA 2015;314:2384–400. 26647260 10.1001/jama.2015.16353

[77] Hsu D Brieva J Silverberg JI. Costs of care for hospitalization for pemphigus in the United States. JAMA Dermatol 2016;152:645–54. 26865293 10.1001/jamadermatol.2015.5240

[78] Chiu HY Chang CJ Lin YJ. National trends in incidence, mortality, hospitalizations, and expenditures for pemphigus in Taiwan. J Dermatol Sci 2020;99:203–8. 32859457 10.1016/j.jdermsci.2020.08.002

[79] Ständer S Färber B Radeke S. Assessment of healthcare costs for patients with pemphigus and bullous pemphigoid in an academic centre in Germany. Br J Dermatol 2020;182:1296–7. 31749141 10.1111/bjd.18731

[80] Brodszky V Tamási B Hajdu K. Disease burden of patients with pemphigus from a societal perspective. Expert Rev Pharmacoecon Outcomes Res 2021;21:77–86. 31978314 10.1080/14737167.2020.1722104

[81] Altman EM. Novel therapies for pemphigus vulgaris. Am J Clin Dermatol 2020;21:765–82. 32860200 10.1007/s40257-020-00544-w

[82] Daoud YJ Amin KG. Comparison of cost of immune globulin intravenous therapy to conventional immunosuppressive therapy in treating patients with autoimmune mucocutaneous blistering diseases. Int Immunopharmacol 2006;6:600–6. 16504922 10.1016/j.intimp.2005.11.002

[83] Hébert V Vermeulin T Tanguy L. Comparison of real costs in the French healthcare system in newly diagnosed patients with pemphigus for first-line treatment with rituximab vs. standard corticosteroid regimen: data from a national multicentre trial. Br J Dermatol 2020;183:121–7. 31657454 10.1111/bjd.18563

[84] Heelan K Hassan S Bannon G. Cost and resource use of pemphigus and pemphigoid disorders pre- and post-rituximab. J Cutan Med Surg 2015;19:274–82. 25775641 10.2310/7750.2014.14092

[85] Das S Agarwal K Singh S. A comparative study to evaluate the efficacy and cost of rituximab versus dexamethasone cyclophosphamide pulse in patients of pemphigus vulgaris. Indian J Dermatol 2021;66:223. 10.4103/ijd.IJD_306_20 PMC8208287 34188295

[86] Saha M Bhogal B Black MM. Prognostic factors in pemphigus vulgaris and pemphigus foliaceus. Br J Dermatol 2014;170:116–22. 24102444 10.1111/bjd.12630

[87] Ljubojević S Lipozencić J Brenner S. Pemphigus vulgaris: a review of treatment over a 19-year period. J Eur Acad Dermatol Venereol 2002;16:599–603. 12482043 10.1046/j.1468-3083.2002.00504.x

[88] Le Hénaff Y Héas S. Individuals with the rare disease pemphigus: a quest for diagnostic. Qual Health Res 2019;29:889–99. 30296923 10.1177/1049732318803590

[89] Daltaban O Özçentik A Akman Karakaş A. Clinical presentation and diagnostic delay in pemphigus vulgaris: a prospective study from Turkey. J Oral Pathol Med 2020;49:681–6. 32516514 10.1111/jop.13052

[90] Hassona Y Cirillo N Taimeh D. Diagnostic patterns and delays in autoimmune blistering diseases of the mouth: a cross-sectional study. Oral Dis 2018;24:802–8. 29383799 10.1111/odi.12839

[91] Sirois DA Fatahzadeh M Roth R. Diagnostic patterns and delays in pemphigus vulgaris: experience with 99 patients. Arch Dermatol 2000;136:1569–70. 11115183 10.1001/archderm.136.12.1569

[92] Sultan AS Villa A Saavedra AP. Oral mucous membrane pemphigoid and pemphigus vulgaris-a retrospective two-center cohort study. Oral Dis 2017;23:498–504. 28084005 10.1111/odi.12639

[93] Shah AA Seiffert-Sinha K Sirois D. Development of a disease registry for autoimmune bullous diseases: initial analysis of the pemphigus vulgaris subset. Acta Derm Venereol 2015;95:86–90. 24691863 10.2340/00015555-1854

[94] Okcin F Ugur O. What does it mean to be pemphigus patient? A qualitative study. Clin Nurs Res 2021;30:790–8. 33764220 10.1177/10547738211002354

[95] Wang EQ Castrillón Velásquez MA Murrell DF. The effects of autoimmune blistering diseases on work productivity: a review. Int J Womens Dermatol 2018;4:131–8. 30175214 10.1016/j.ijwd.2017.11.001 PMC6116828

[96] Wang EQ Radjenovic M Castrillón MA. The effect of autoimmune blistering diseases on work productivity. J Eur Acad Dermatol Venereol 2018;32:1959–1966. 29730897 10.1111/jdv.15062

[97] Hsu DY Brieva J Sinha AA. Comorbidities and inpatient mortality for pemphigus in the U.S.A. Br J Dermatol 2016;174:1290–8. 26864457 10.1111/bjd.14463

[98] Hsu DY Brieva J Nardone B. Association of pemphigus and systemic corticosteroid use with comorbid health disorders: a case-control study. Dermatol Online J 2017;23. 29447647

[99] Czerninski R Zadik Y Kartin-Gabbay T. Dietary alterations in patients with oral vesiculoulcerative diseases. Oral Surg Oral Med Oral Pathol Oral Radiol 2014;117:319–23. 24144994 10.1016/j.oooo.2013.08.006

[100] Le Hénaff Y Héas S. Engagement in leisure and physical activities: analysing the biographical disruptions of a rare chronic disease in France. Sociol Health Illn 2020;42:65–79. 31498461 10.1111/1467-9566.12987

[101] Momtazbakhsh M Zarei A Ashraf Fanjouie F Daneshpazhooh M. The effect of leisure time physical activities on quality of life and clinical symptoms of pemphigus vulgaris. Iranian J Nurs Res 2020;15:29–39.

[102] Akhyani M Chams-Davatchi C Naraghi Z. Cervicovaginal involvement in pemphigus vulgaris: a clinical study of 77 cases. Br J Dermatol 2008;158:478–82. 18070212 10.1111/j.1365-2133.2007.08356.x

[103] Piri F Firouzkouhi M Abdollahimohammad A. Exploring pemphigus challenges based on the patient experiences: a descriptive phenomenological research. Prensa Med Argent 2017;103:6.

[104] Sajedianfard S Handjani F Saki N. Family dermatology life quality index in patients with pemphigus vulgaris: a cross-sectional study. Indian J Dermatol Venereol Leprol 2021;87:375–8. 31464197 10.4103/ijdvl.IJDVL_276_18

[105] Ghodsi SZ Asadi A Ghandi N. Family impact of pemphigus disease in an Iranian population using the Family Dermatology Life Quality Index. Int J Womens Dermatol 2020;6:409–13. 33898709 10.1016/j.ijwd.2020.09.004 PMC8060665

[106] Ghasemi M Alhani F Gholami M. The effect of family-centered empowerment model on quality of life of female patients with pemphigus vulgaris referred to Razi hospital. IJNR 2018;13:1–10.

[107] Terrab Z Benchikhi H Maaroufi A. Quality of life and pemphigus. Ann Dermatol Venereol 2005;132:321–8. 15886558 10.1016/s0151-9638(05)79276-0

[108] Rencz F Gulácsi L Tamási B. Health-related quality of life and its determinants in pemphigus: a systematic review and meta-analysis. Br J Dermatol 2015;173:1076–80. 25894470 10.1111/bjd.13848

[109] Mayrshofer F Hertl M Sinkgraven R. Significant decrease in quality of life in patients with pemphigus vulgaris. Results from the German Bullous Skin Disease (BSD) Study Group. Journal der Deutschen Dermatologischen Gesellschaft = J German Soc Dermatol: JDDG 2005;3:431–5. 10.1111/j.1610-0387.2005.05722.x 15892845

[110] Ghodsi SZ Chams-Davatchi C Daneshpazhooh M. Quality of life and psychological status of patients with pemphigus vulgaris using Dermatology Life Quality Index and General Health Questionnaires. J Dermatol 2012;39:141–4. 21967321 10.1111/j.1346-8138.2011.01382.x

[111] Mitev A Rencz F Tamási B. Subjective well-being in patients with pemphigus: a path analysis. Eur J Health Economics 2019;20:101–7. 10.1007/s10198-019-01067-w PMC6544602 31098885

[112] Sebaratnam DF Hanna AM Chee SN. Development of a quality-of-life instrument for autoimmune bullous disease: the Autoimmune Bullous Disease Quality of Life questionnaire. JAMA Dermatol 2013;149:1186–91. 23925444 10.1001/jamadermatol.2013.4972

[113] Sebaratnam DF Okawa J Payne A. Reliability of the autoimmune bullous disease quality of life (ABQOL) questionnaire in the USA. Qual Life Res 2015;24:2257–60. 25795375 10.1007/s11136-015-0965-z PMC4767525

[114] Kalinska-Bienias A Jakubowska B Kowalewski C. Measuring of quality of life in autoimmune blistering disorders in Poland. Validation of disease - specific Autoimmune Bullous Disease Quality of Life (ABQOL) and the Treatment Autoimmune Bullous Disease Quality of Life (TABQOL) questionnaires. Adv Med Sci 2017;62:92–6. 28208086 10.1016/j.advms.2016.07.002

[115] Yang B Chen G Yang Q. Reliability and validity of the Chinese version of the autoimmune bullous disease quality of life (ABQOL) questionnaire. Health Qual Life Outcomes 2017;15:31. 28153023 10.1186/s12955-017-0594-z PMC5290597

[116] Saleh MA Zaraa I Doss N. Assessment of the quality of life of Egyptian and Tunisian autoimmune bullous diseases’ patients using an Arabic version of the autoimmune bullous disease quality of life and the treatment of autoimmune bullous disease quality of life questionnaires. Anais brasileiros de dermatologia 2019;94:399–404. 31644610 10.1590/abd1806-4841.20197198 PMC7007032

[117] Teimourpour A Hedayat K Salarvand F. Autoimmune Bullous Disease Quality of Life (ABQoL) questionnaire: validation of the translated Persian version in pemphigus vulgaris. Int J Women’s Dermatol 2020;6:306–10. 33015292 10.1016/j.ijwd.2020.03.043 PMC7522916

[118] Patsatsi A Kokolios M Kyriakou A. Quality of life in Greek patients with autoimmune bullous diseases assessed with ABQOL and TABQOL indexes. Acta Derm Venereol 2017;97:1145–7. 28660281 10.2340/00015555-2737

[119] Ferries L Gillibert A Duvert-Lehembre S. Sensitivity to change and correlation between the autoimmune bullous disease quality-of-life questionnaires ABQOL and TABQOL, and objective severity scores. Br J Dermatol 2020;183:944–5. 32374931 10.1111/bjd.19173

[120] Tjokrowidjaja A Daniel BS Frew JW. The development and validation of the treatment of autoimmune bullous disease quality of life questionnaire, a tool to measure the quality of life impacts of treatments used in patients with autoimmune blistering disease. Br J Dermatol 2013;169:1000–6. 24102329 10.1111/bjd.12623

[121] Behkar A Garmaroudi G Nasimi N. Assessing Quality of Life in Patients with Autoimmune Bullous Diseases Using the Persian Version of Treatment of Autoimmune Bullous Disease Quality of Life (TABQOL) Questionnaire finds similar effects in women as men. Int J Women’s Dermatol 2021;8:e004. 10.1097/JW9.0000000000000004 PMC9112387 35620025

[122] Chen G Yang B Zhang Z. Chinese version of the treatment of autoimmune bullous disease quality of life questionnaire: reliability and validity. Indian J Dermatol Venereol Leprol 2018;84:431–6. 28485307 10.4103/ijdvl.IJDVL_538_16

